# Supplementary material for: Somatically mutated ABL1 is an actionable and essential NSCLC survival gene
Source: EMBO Mol Med. 2016 Jan 12;8(2):105–16. doi: 10.15252/emmm.201505456 (PMC4734836; doi:10.15252/emmm.201505456)
Supplement: Supplementary file 1 — Appendix [file EMMM-8-105-s001.pdf]

Somatically Mutated ABL1 Represents an Actionable and Essential Driver of NSCLC Cancer

Ewelina Testoni[1], Natalie L. Stephenson[1*], Pedro Torres-Ayuso[1*], Anna A. Marusiak[1], Eleanor W. Trotter[1], Andrew Hudson[1], Cassandra L. Hodgkinson[2], Christopher J. Morrow[2], Caroline Dive[2] and John Brognard[1**]

[1]Signalling Networks in Cancer Group, [2] Clinical and Experimental Pharmacology Group, Cancer Research UK Manchester Institute, The University of Manchester, Manchester, UK.

[*] Indicates equal contribution.

[**]To whom correspondence should be addressed.

Table of Contents

Appendix materials and methods 2

Appendix Figures 5

Appendix Figure S1 5

Appendix Figure S2 6

Appendix Figure S3 7

Appendix Figure S4 8

Appendix Figure S5 9

Appendix Figure S6 10

Appendix Figure S7 11

Appendix Figure S8 12

Appendix Figure S9 13

Appendix Figure S10 14

Appendix Figure S11 15

Appendix Tables 16

Appendix Table S1 16

Appendix Table S2 17

Appendix References 18

Appendix materials and methods

Cell line sequencing

Total DNA was isolated from H1915 and H2110 cells using DNeasy Blood and Tissue DNA extraction kit (Qiagen). Forward primer (5'-aacgcacggacatcaccatgaa-3') and reverse primer (5'-ggccaaaatcagctaccttcacca-3') for amplification of Exon 6 in ABL1 gene were designed using Aceview database and AmplifX1.5.4 Primer Design Program. Exon 6 was reverse transcribed and amplified using Phusion High-Fidelity PCR master mix with HF Buffer (New England BioLabs), according to manufacturer's protocol. The PCR reaction was purified using Illustra ExoStar enzymatic PCR and sequencing clean up reaction kit (GE Healthcare) and the resulting product subjected to Sanger sequencing with sequencing primer (5'-ggacaccatggaggtggaa-3').

Site-directed mutagenesis and cloning

The primers were designed using Agilent's QuickChange Primer Design Program and purchased from Eurofins MWG. The primer sequences are listed below. Mutations were introduced into the plasmids pDONR223-ABL1 (Addgene, 23939) using the QuikChange II kit (Agilent). The plasmid DNA was purified using QIAprep Spin MiniPrep Kit (Qiagen) and the presence of the mutations confirmed by Sanger sequencing (conducted by Molecular Biology Core Facility at the Cancer Research UK Manchester Institute). pcDNA3HA, converted into Gateway destination vector (kind gift from Prof. Alexandra Newton), was used to clone the mutated and the wild type ABL1 genes from pDONR223 using the LR clonase reaction (Gateway® Technology, Invitrogen). The ABL1 constructs were then transformed into One Shot® OmniMAX™ 2 T1^R *E. coli* cells (Invitrogen) and the DNA constructs were purified using Plasmid Plus Maxi Kit (Qiagen). A pcDNA3.1(+) vector (Invitrogen) was used as an empty vector control where required.

The following primers were designed using Agilent's QuickChange Primer Design Program and purchased from Eurofins MWG.ABL1 R351W forward 5'-tgagggagtgcaactggcaggaggtga ac-3'

ABL1 R351W reverse 5'-gttcacctcctgccagttgcactccctca-3'

ABL1 G340L forward 5'-tcactgagttcatgacctacctaaacctcctggactacctgag-3'

ABL1 G340L reverse 5'-ctcaggtagtccaggaggtttaggtaggtcatgaactcagtga-3'

ABL2 W469C forward 5'-caattaaatctgacgtctgcgcttttggggtattgttgt-3'

ABL2 W469C reverse 5'-acaacaataccccaaaagcgcagacgtcagatttaattg-3'

ABL2 Y399C forward 5'-cagatttcttctgcaatggagtgcttagagaagaagaatttcatc-3'

ABL2 Y399C reverse 5'-gatgaaattcttcttctctaagcactccattgcagaagaaatctg-3'

ABL2 G300V forward 5'-ggggcggtcagtatgtagaggtttacgttgg-3'

ABL2 G300V reverse 5'-ccaacgtaaacctctacatactgaccgcccc-3'

ABL1 T315I forward 5'-agcccccgttctatatcatcattgagttcatgacc-3'

ABL1 T315I reverse 3'-tcgggggcaagatatagtagtaactcaagtactgg-5'

ABL1 R47G forward 3'-ctgagtgaagccgctggttggaactccaagg-5'

ABL1 R47G reverse ccttggagttccaaccagcggcttcactcag

ABL1 R732S forward 3'-gacacggagtggagttcagtcacgctgcc-5'

ABL1 R732S reverse 3'-ggcagcgtgactgaactccactccgtgtc-5'

ABL1 P806L forward 3'-ggagtccagcctgggctccagcc-5'

ABL1 P806L reverse 3'-ggctggagcccaggctggactcc-5'

ABL1 R166K forward 3'-ggtccatctcgctgaaatacgaagggagggt-5'

ABL1 R166K reverse 3'-accctcccttcgtatttcagcgagatggacc-5'

ABL1 A137S forward 3'-tgtgtcccgcaatgccagtgagtatctgctgagc-5'

ABL1 A137S reverse 3'-gctcagcagatactcactggcattgcgggacaca-5'

ABL1 I242M forward 3'-gatggaacgcacggacatgaccatgaagcacaa-5'

ABL1 I242M reverse 3'-ttgtgcttcatggtcatgtccgtgcgttccatc-5'

ABL1 A137S forward 3'-tgtgtcccgcaatgccagtgagtatctgctgagc-5'

ABL1 A137S reverse 3'-gctcagcagatactcactggcattgcgggacaca-5'

ABL1 I242M forward 3'-gatggaacgcacggacatgaccatgaagcacaa-5'

ABL1 I242 reverse 3'-ttgtgcttcatggtcatgtccgtgcgttccatc-5'

Structural modeling

Homology models of R351W and G340L tyrosine kinase domains of ABL1 were based on the crystal structures of ABL1 kinase domain in the inactive (pdb 1IEP-3) and active (pdb 2GQG) conformations. Further homology models of ABL1 protein with R351W and G340L mutations were created based on the crystal structure of ABL1 SH3, SH2 and kinase domain (pdb 10PL-1). The homology models were created using SwissModel (Arnold et al, 2006). Molecular dynamics simulations were performed using GROMACS version 4.5.3 using the GROMOS96 53A6 force field parameter set. All titratable amino acids were assigned their canonical state at physiological pH, short-range interactions were cut off at 1.4 nm and long-range

electrostatics were calculated using the particle mesh Ewald summation (Essmann et al, 1995). Dispersion correction was applied to energy and pressure terms accounting for truncation of van der Waals forces and periodic boundary conditions were applied in all directions. Protein constructs were placed in a cubic box of 100 nM NaCl in simple point charge water with at least 1 nm distance between the protein construct and box edge in all directions. Neutralizing counter ions were added and steepest decent energy minimization was performed, followed by a two-step NVT/NPT equilibration. Both equilibration steps maintained a constant number of particles and temperature, NVT equilibration was performed for 100 ps maintaining a constant volume, followed by 100 ps of NPT equilibration maintaining a constant pressure. Temperature was maintained at 37°C by coupling protein and non-protein atoms to separate temperature coupling baths (Berendsen et al, 1984), pressure was maintained at 1.0 bar (weak coupling). All position restraints were then removed and simulations were performed for 5 ns using the Nose-Hoover thermostat (Nose, 1984) and the Parrinello-Rahman barostat (Parrinello & Rahman, 1981). RMSD analysis compared the position of specified residues within the starting structure to their position at each time point to determine how the positions of these residues changed over time. Simulations were performed in triplicate; values shown in figures are averages with standard error of the mean highlighted as error bars. Images were created using PyMol version 1.5.0.5.

Appendix Figures

Appendix Figure S1

A

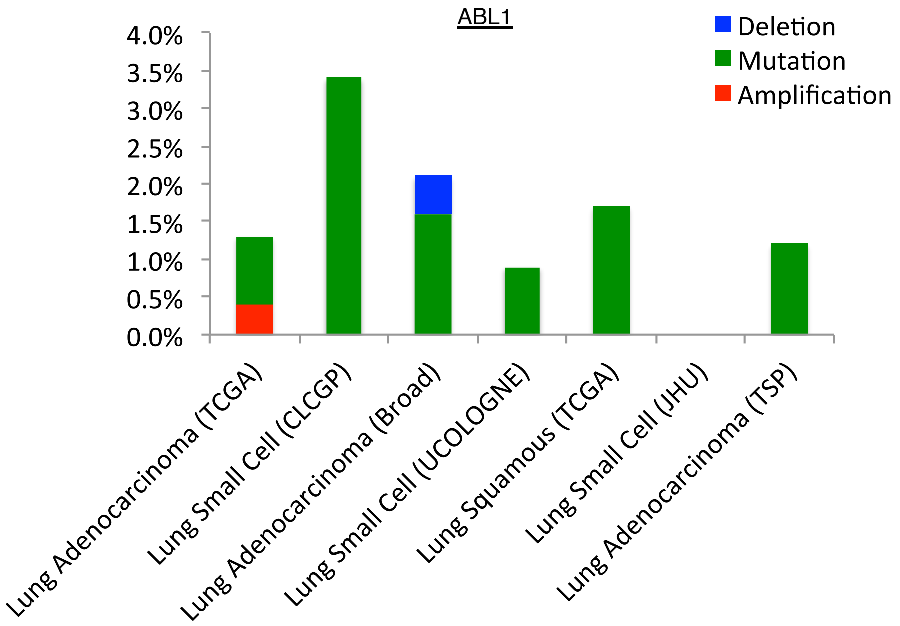

B

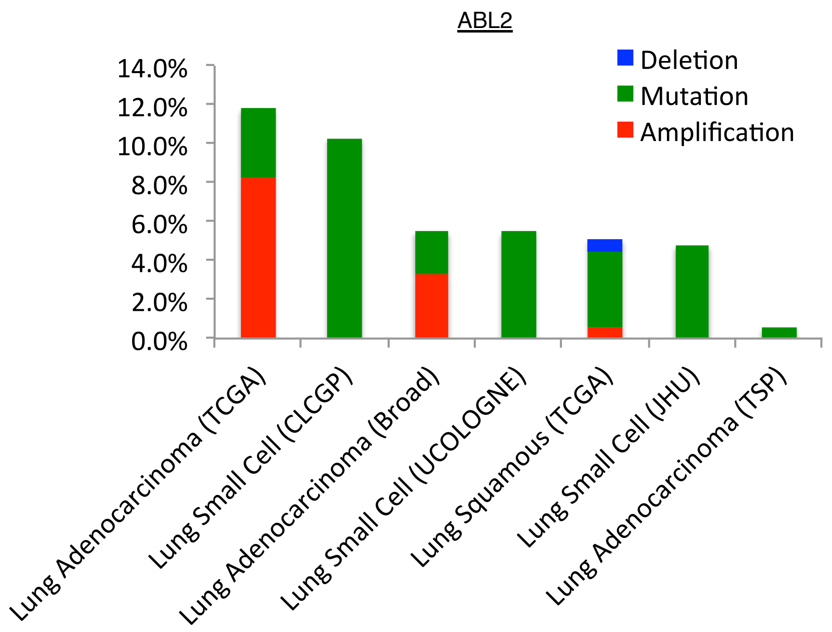

(A) ABL1 and (B) ABL2 gene alterations in lung cancer datasets available on cBio Cancer Genomic Portal (Cerami, Gao et al., 2012).

Appendix Figure S2

A

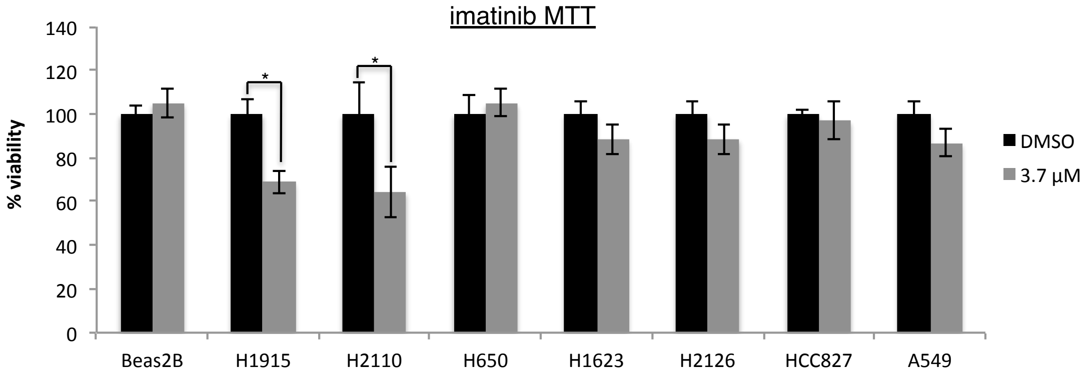

B

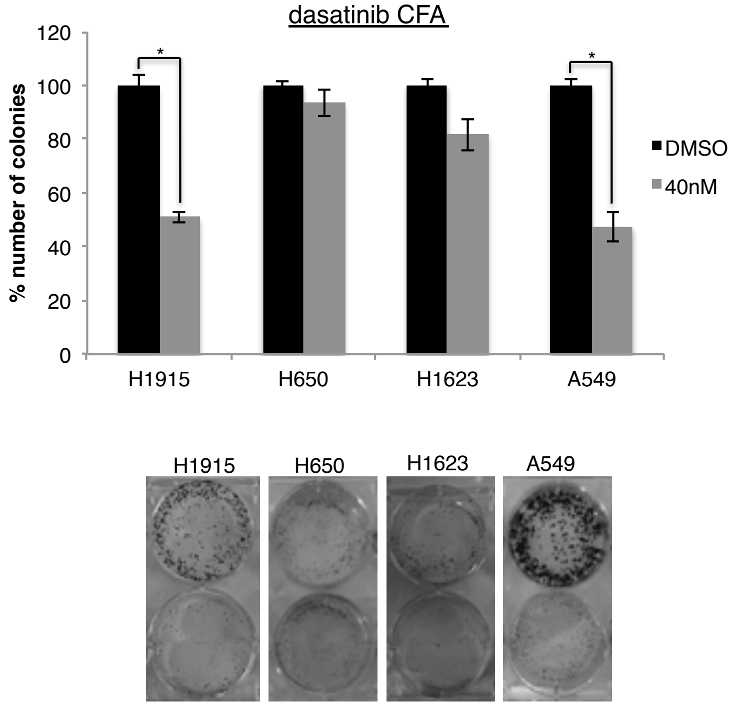

(A) MTT assay for NSCLC cell lines and Beas-2B cells treated with imatinib or DMSO. Data are presented as mean ±S.E.M., n=9, *P < 0.001 by two-tailed unpaired Student's t-test. (B) Colony formation assay (CFA) with dasatinib with representative images of colonies formed by each cell line within the panel. Data are presented as mean ±S.E.M., n=6, *P < 0.001 by two-tailed unpaired Student's t-test.

Appendix Figure S3

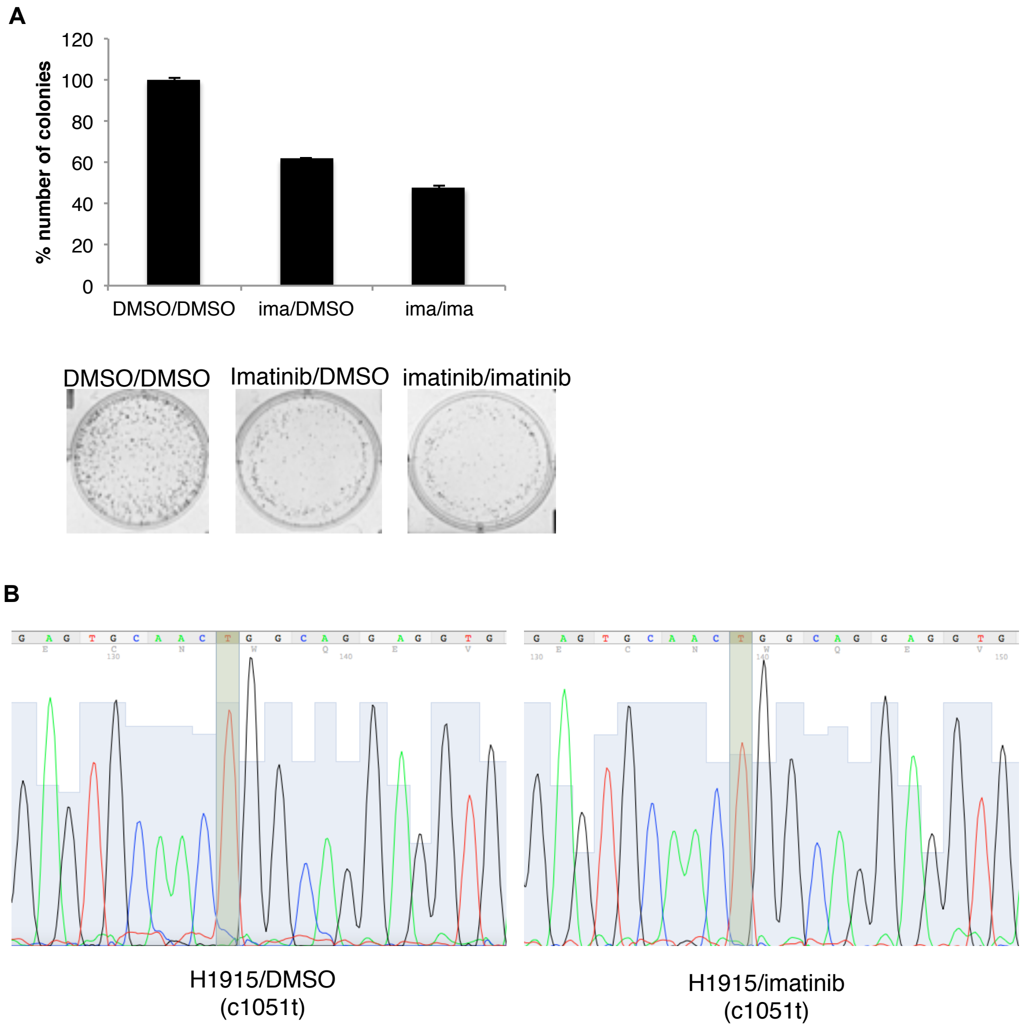

H1915 show increased imatinib sensitivity and reduced colony forming potential after repeated exposure to the drug. (A) Colony formation assay (CFA) for H1915 cells re-plated from cells previously treated with either DMSO or 2μM imatinib. DMSO/DMSO, cells re-plated from DMSO treatment and re-treated with DMSO; ima/DMSO, cells re-plated from treatment with 2μM imatinib and re-treated with DMSO; ima/ima, cells re-plated from 2μM imatinib treatment and re-treated with 2μM imatinib. Error bars represent ±S.D., n=3. The figure shows the representative images of colonies formed from the re-plated cells. (B) Sanger sequencing of DNA samples collected from H1915 cells treated with DMSO or imatinib. The data indicate the presence of C1051T point mutation (R351W) in both H1915/DMSO and H1915/imatinib cells.

Appendix Figure S4

A

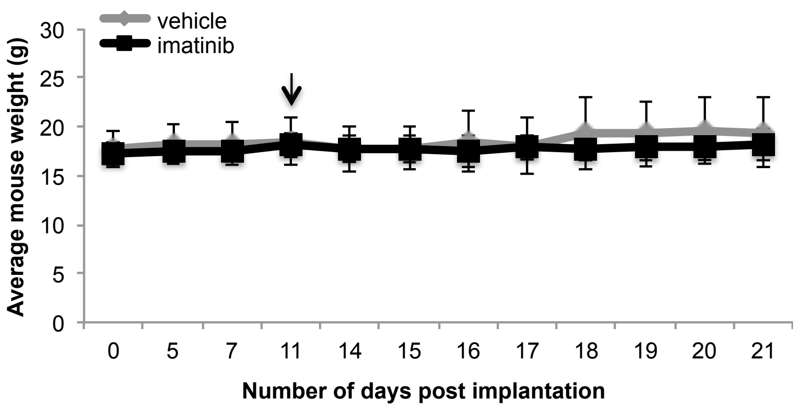

B

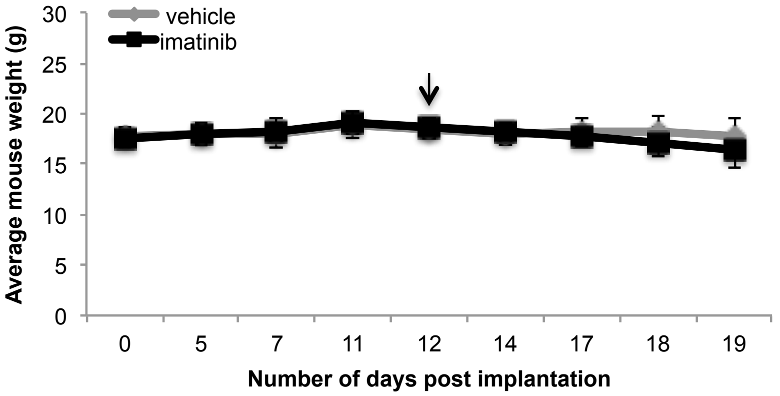

C

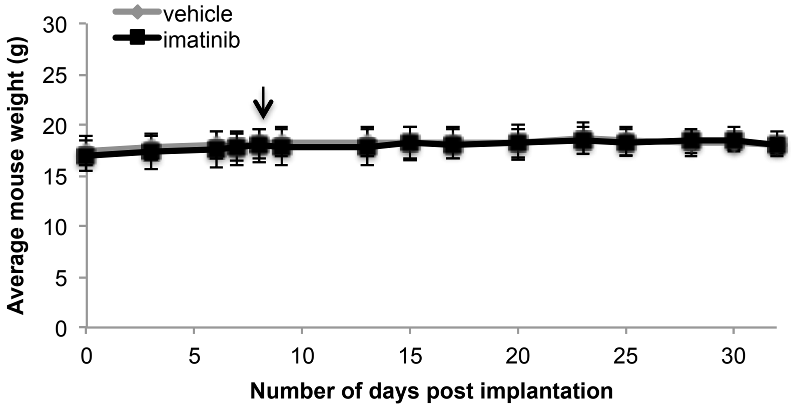

The body weights of mice with H1915, H650 and H1915-ABL1^{T513I} xenografts are not affected by the treatment with imatinib. Average weights of tumour bearing mice derived from H1915 (A) H650 (B) and H1915 with doxycycline inducible ABL1 with gatekeeper mutation (C) cells. The mice weight was monitored 3 times a week from the day of implantation. Arrows indicate the first day of the treatment with either imatinib (100mg/kg, twice daily) or vehicle. Data are presented as mean ±S.D., n= 6-10 mice per group.

Appendix Figure S5

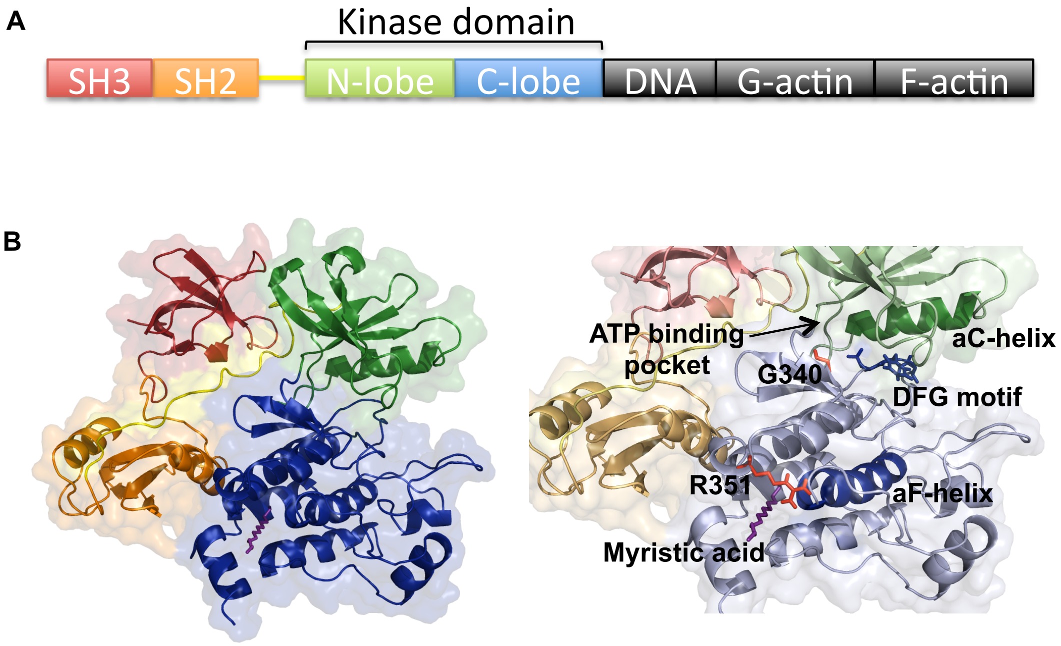

(A) Schematic showing simplified domain composition of the ABL1/ABL2 proteins. (B) Structural composition of the SH3, SH2 and kinase domains of ABL1 with key regions highlighted. The figure indicates the position of R351 and G340 within the kinase domain.

Appendix Figure S6

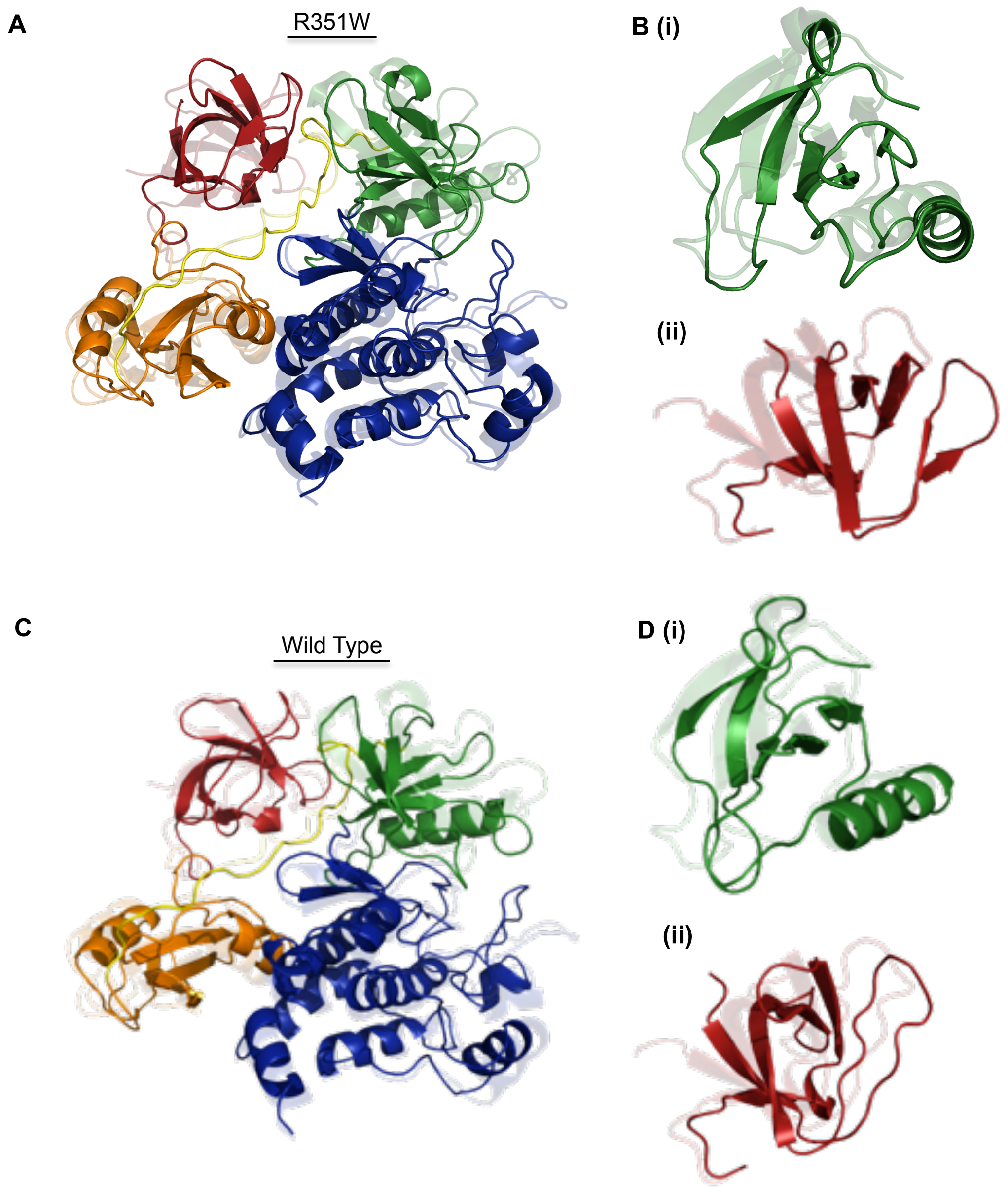

Wide scale destabilisation is observed within R351W mutant ABL1 during molecular dynamics simulations. Molecular dynamics simulations performed on ABL1 constructs. Images show the start (t = 0 ps, light image) and end (t = 5000 ps, dark image) positions of the simulated structures for R351W (A and B) compared to wild type (C and D). A large degree of movement is observed within the SH3 domain (red, B (i)) and the N-lobe of the kinase domain (green, B (ii)) in the construct that harbours the R351W mutation compared to the wild type structure, where little movement is observed in these structures (D (i) and (ii)). Increased movement within the SH3 domain could suggest a destabilization of the autoinhibited conformation allowing for increased kinase activity.

Appendix Figure S7

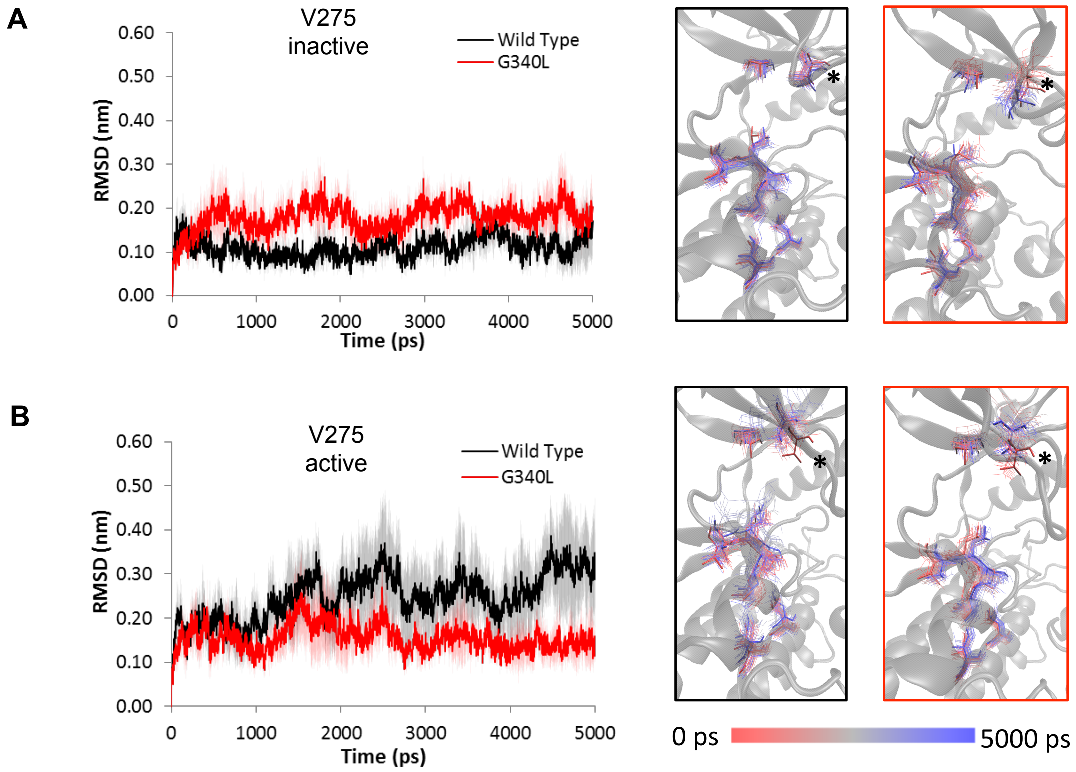

Structural analysis and molecular dynamics simulations indicate that G340L mutation in ABL1 causes stabilization of the C-spine within the active kinase domain. Molecular dynamics simulations performed on ABL1 constructs as described previously. Root-mean-squared deviation (RMSD) analysis shows movement in the V275 residue within the C-spine of the inactive (A) and active (B) conformations of ABL1 in wild type and G340L constructs. Images show time lapse changes of C-spine residues in wild type (black outline) and G340L (red outline) constructs (residue V275 highlighted with asterisk).

Appendix Figure S8

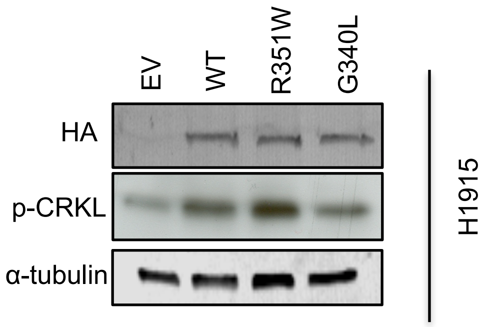

R351W but not G340L mutations in ABL1 enhance CRKL phosphorylation. Western blot analysis of transient overexpression of empty vector (EV), wild type (WT) and mutant ABL1 constructs (R351W and G340L) in H1915 cells.

Appendix Figure S9

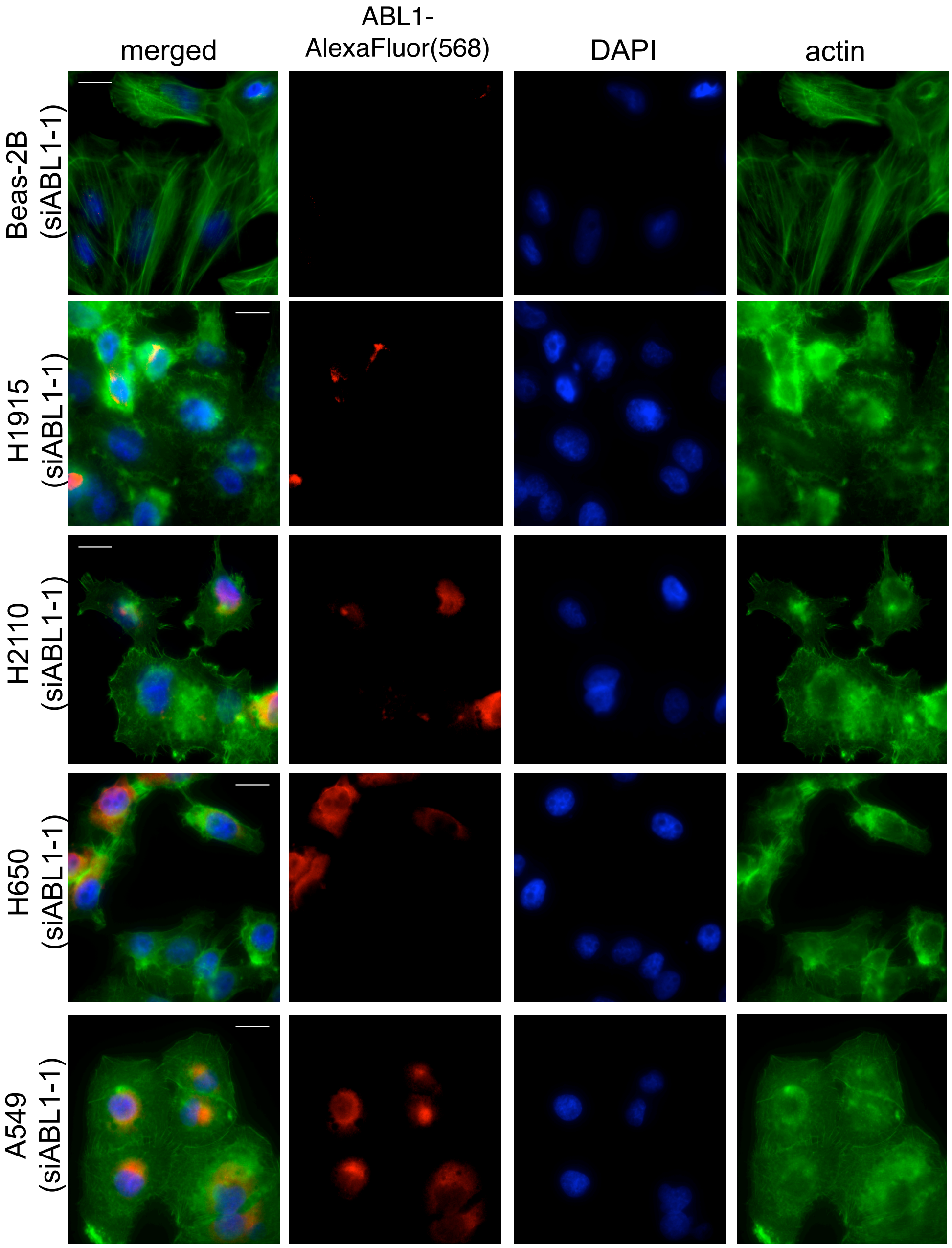

Immunofluorescence imaging of ABL1 in the panel of NSCLC cell lines transfected with ABL1 siRNA as a control for the endogenous ABL1 staining shown on figure 5. siABL1-1 was used for the effective ABL1 knockdown, as verified in Figs 2D-F and EV3A. Images were taken using the same settings as for the images shown on figure 5. Scale bar = 20 μm.

Appendix Figure S10

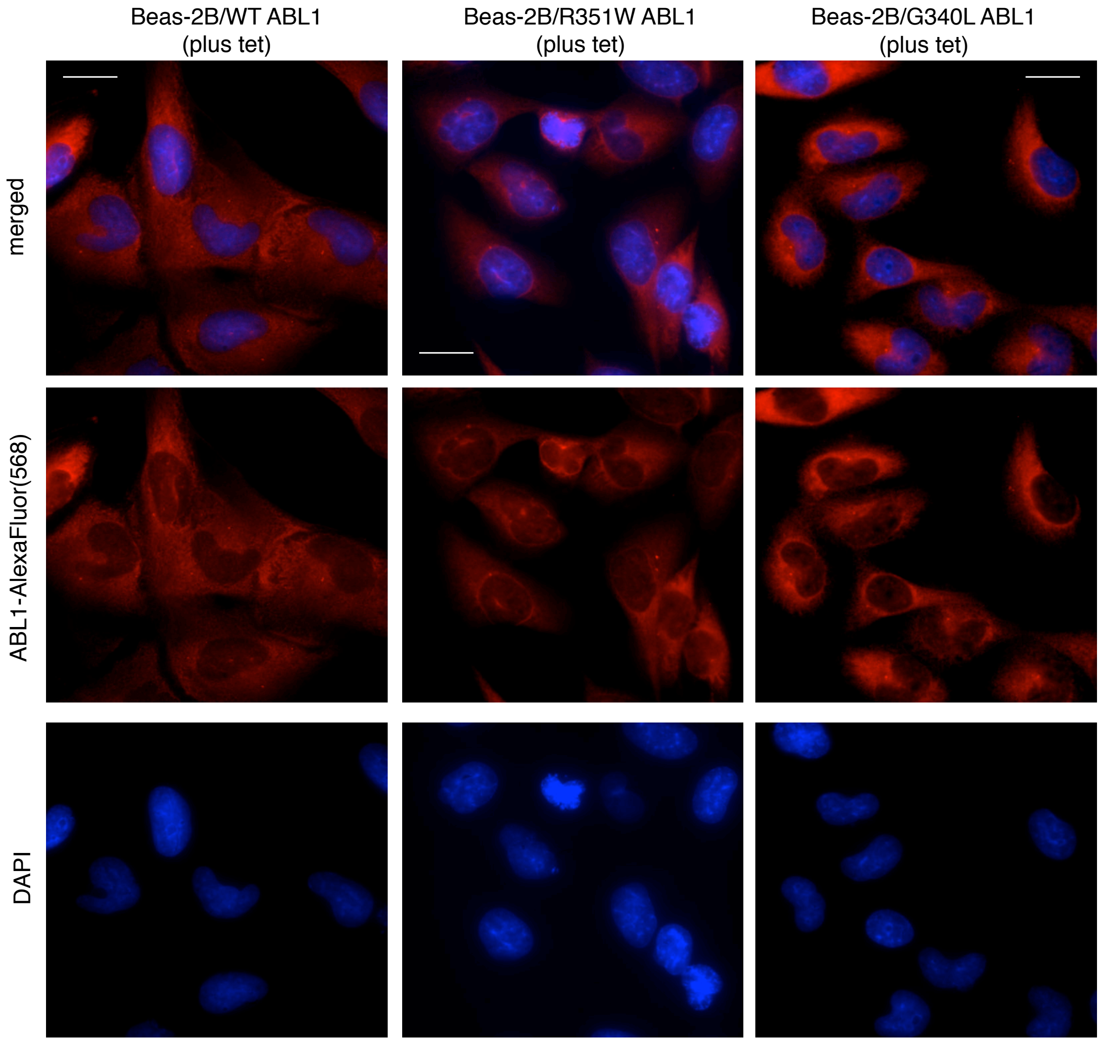

G340L and R351W ABL1 mutants show increased cytoplasmic localization compared to WT ABL1. Immunofluorescence imaging of tetracycline induced overexpression of wild type ABL (WT) and mutant ABL proteins (R351W and G340L) in inducible Beas-2B cell lines. Scale bar = 20 μm.

Appendix Figure S11

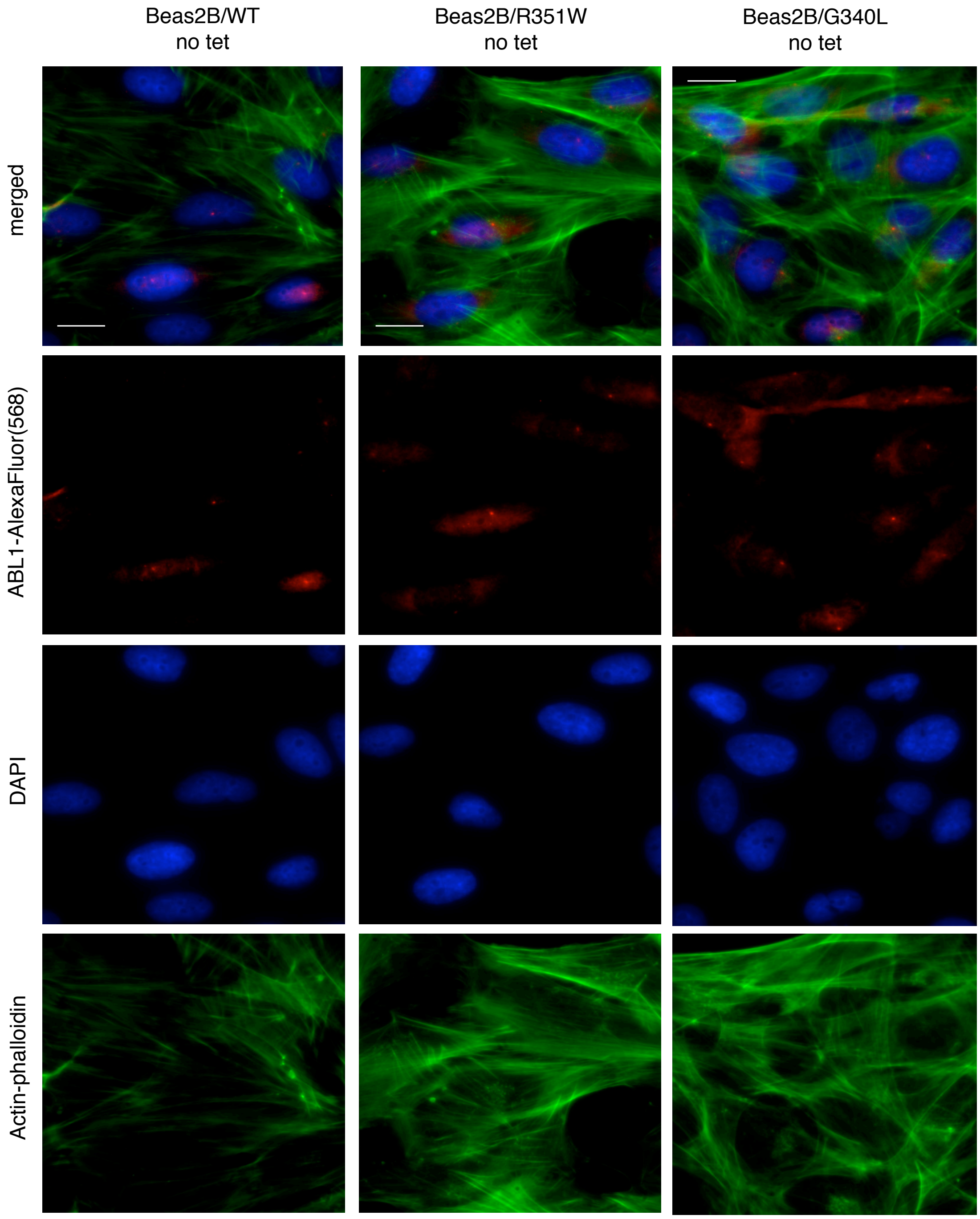

Immunofluorescence imaging of endogenous ABL1 in inducible Beas-2B cell lines without tetracycline induction. Images were taken using the same settings as for the overexpressed ABL1 in tetracycline treated cells shown in Figure S10 for direct comparison of ABL1 staining in induced versus un-induced cells. Phalloidin staining of actin cytoskeleton was used as positive control. Scale bar = 20 µm.

Appendix Tables

Appendix Table S1

Computational predictions of point mutations identified in NSCLC cell lines.

Mutation (cell line)	Zygosity	Mutation assessor	pMut	Polyphen 2
ABL1 G340L (H2110)	hetero-zygous	N/A	pathological	probably damaging
ABL1 R351W (H1915)	homo-zygous	medium	pathological	probably damaging
ABL2 Y399C (H1623)	hetero-zygous	high	neutral	probably damaging
ABL2 W469C (H650)	homo-zygous	high	neutral	N/A

Computational predictions on the functional effects of point mutations in ABL1 (G340L and R351W) and ABL2 (Y399C and W469C) using Mutational assessor, pMut, and Polyphen2. Numbering of substituted residues corresponds to isoforms B of both ABL1 and ABL2. Cell lines with the ABL1 and ABL2 mutations were selected for the current study from CCLE database.

Appendix Table S2

Computational predictions of point mutations in ABL1 identified in primary tumours.

Mutation (tumour)	Polyphen 2	Provean	SIFT
ABL1 R47G (lung)	possibly damaging	deleterious	damaging
ABL1 R166K (skin)	benign	deleterious	damaging
ABL1 A137S (lung)	probably damaging	deleterious	damaging
ABL1 I242M (breast)	probably damaging	deleterious	damaging
ABL1 R712S (lung)	probably damaging	neutral	tolerated
ABL1 P806L (lung)	probably damaging	deleterious	damaging

Computational predictions on the functional effects of point mutations in *ABL1* identified in primary tumors using Polyphen2, Provean and SIFT. Numbering of substituted residues corresponds to isoform A of ABL1.

Appendix References

Abraham MJ, Gready JE (2011) Optimization of parameters for molecular dynamics simulation using smooth particle-mesh Ewald in GROMACS 4.5. *J Comput Chem* **32:** 2031-40

Berendsen HJC, Postma JPM, Gunsteren WF, DiNola A, Haak JR (1984) Molecular dynamics with coupling to an external bath. *J Chem Phys* **81:** 3684-90

Cerami E, Gao J, Dogrusoz U, Gross BE, Sumer SO, Aksoy BA, Jacobsen A, Byrne CJ, Heuer ML, Larsson E, Antipin Y, Reva B, Goldberg AP, Sander C, Schultz N (2012) The cBio cancer genomics portal: an open platform for exploring multidimensional cancer genomics data. Cancer Discov **2:** 401-4

Essmann U, Perera L, Berkowitz ML, Darden T, Lee H, Pedersen LG (1995) A smooth particle mesh Ewald method. *J Chem Phys* **103:** 8577-93

Nose S (1984) A unified formulation of the constant temperature molecular dynamics methods. *J Chem Phys* **81:** 511-9

Parrinello M, Rahman A (1981) Polymorphic transitions in single crystals: A new molecular dynamics method. *Journal of Applied Physics* **52:** 7182-90
