# Supplementary material for: Somatically mutated ABL1 is an actionable and essential NSCLC survival gene
Source: EMBO Mol Med. 2016 Jan 12;8(2):105–16. doi: 10.15252/emmm.201505456 (PMC4734836; doi:10.15252/emmm.201505456)
Supplement: Supplementary file 2 — Expanded View Figures PDF [file EMMM-8-105-s002.pdf]

Expanded View Figures

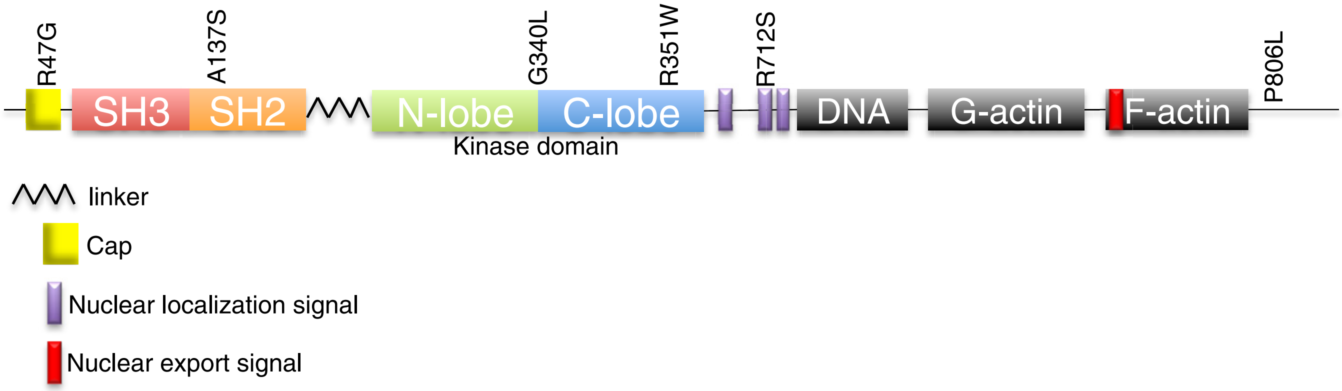

Figure EV1. Somatic ABL1 mutations occur throughout the protein.
Schematic of simplified domain composition of ABL1 with locations of the somatic mutations found in NSCLC cell lines and primary lung tumors indicated.

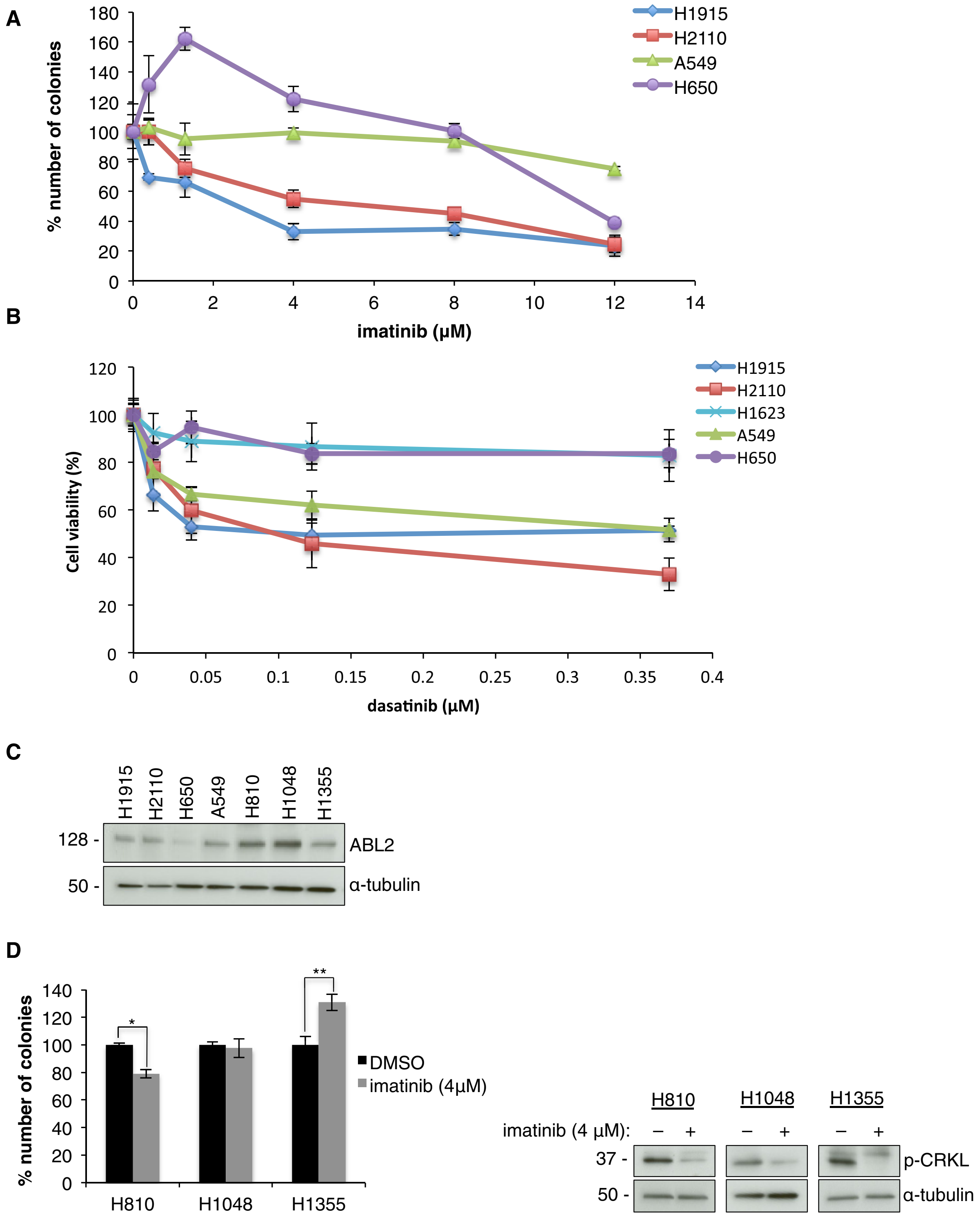

Figure EV2. NSCLC cells with ABL1 mutations are sensitive to clinically relevant concentrations of ABL inhibitors.

A, B Kinetics of imatinib and dasatinib treatment in NSCLC cell lines. Dose–response curves for (A) imatinib and (B) dasatinib treatment determined by (A) colony formation assay and (B) MTT assay in selected lung cancer cell lines. Error bars represent mean ± S.E.M., *n* = 6.

C, D The role of ABL2 amplification in lung cancer cell lines. (C) Western blot showing relative ABL2 protein levels in the panel of lung cancer cell lines. (D) Left, colony formation assay with imatinib in H810, H1048, and H1355. Error bars represent ± S.E.M., *n* = 9, **P* = 6 × 10^{-6}, ***P* = 0.002 by two-tailed unpaired Student's *t*-test. Right, Western blot analysis in the presence of imatinib.

Source data are available online for this figure.

© 2016 The Authors

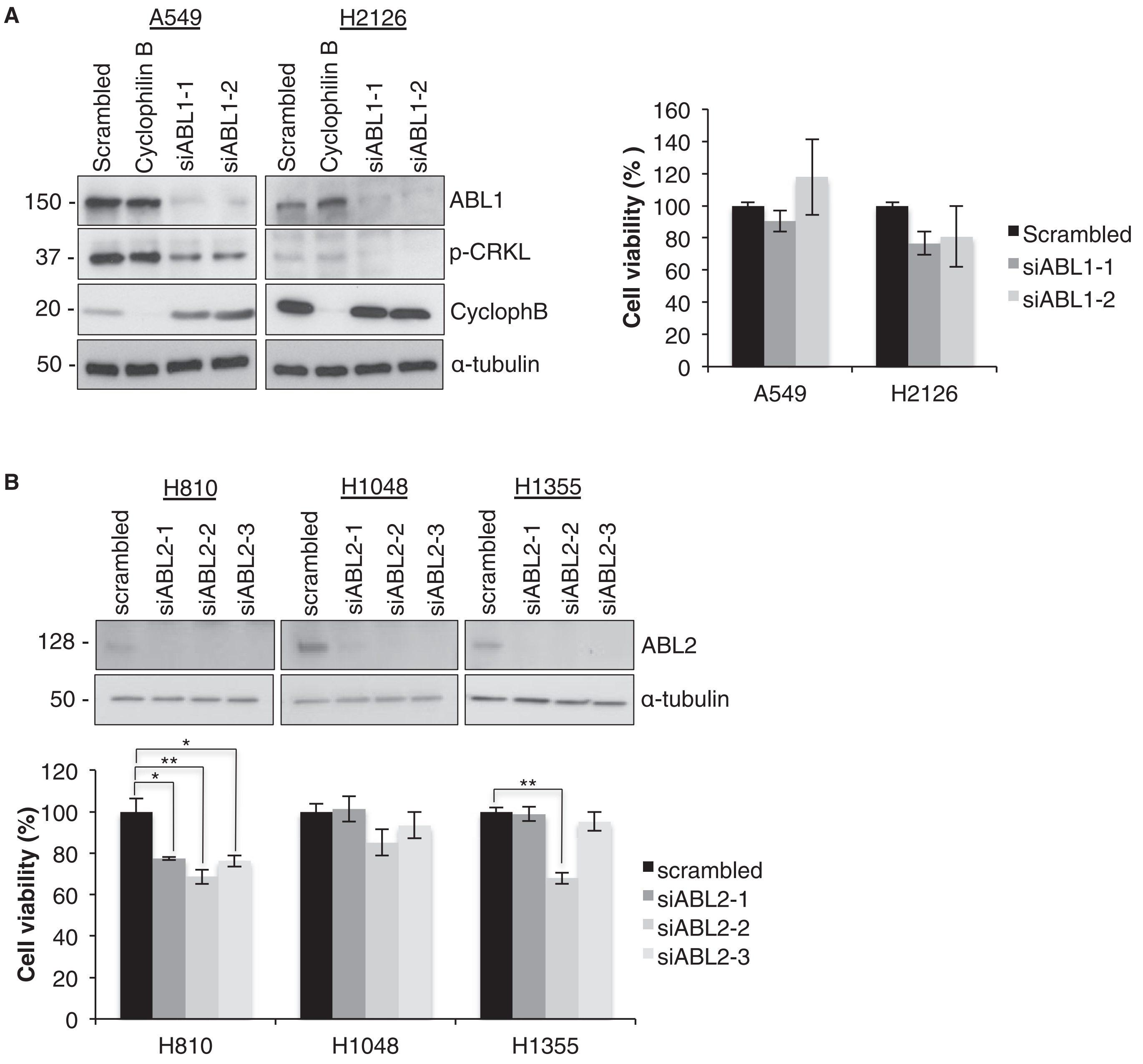

Figure EV3. Wild-type ABL1 or amplified ABL2 are not significant drivers in lung cancer.

A Depletion of ABL1 in NSCLC cells with wild-type ABL1/2 does not reduce cell viability. Left, Western blot analysis of efficient ABL1 knockdown in A549 and H2126 cells transfected with two different ABL1 siRNAs (siABL1-1 and siABL1-2) compared to non-targeting negative control (scrambled) and positive control (Cyclophillin B) siRNAs. Right, MTT assay for A549 and H2126 cells transfected with ABL1 siRNA (siABL1-1 and siABL1-2). Data were normalized to non-targeting siRNA (scrambled). Error bars represent ± S.E.M., n = 9.

B Depletion of ABL2 has minor effect on cells with ABL2 amplification. Top, Western blot analysis and (bottom) MTT assay for ABL2 knockdown in lung cancer cells with ABL2 amplification (H810, H1048, and H1355) transfected with two different ABL2 siRNAs (siABL2-1 and siABL2-2) compared to non-targeting negative control (scrambled). Error bars represent mean ± S.E.M., n = 9, *P < 0.005, **P < 0.001 by two-tailed unpaired Student's t-test (H810, scrambled vs. siABL2-1 P = 0.003, vs. ABL2-si2 P = 0.0008, vs. siABL2-3 P = 0.003; H1355, scrambled vs. siABL2-2 $P = 5 \times 10^{-8}$).

Source data are available online for this figure.

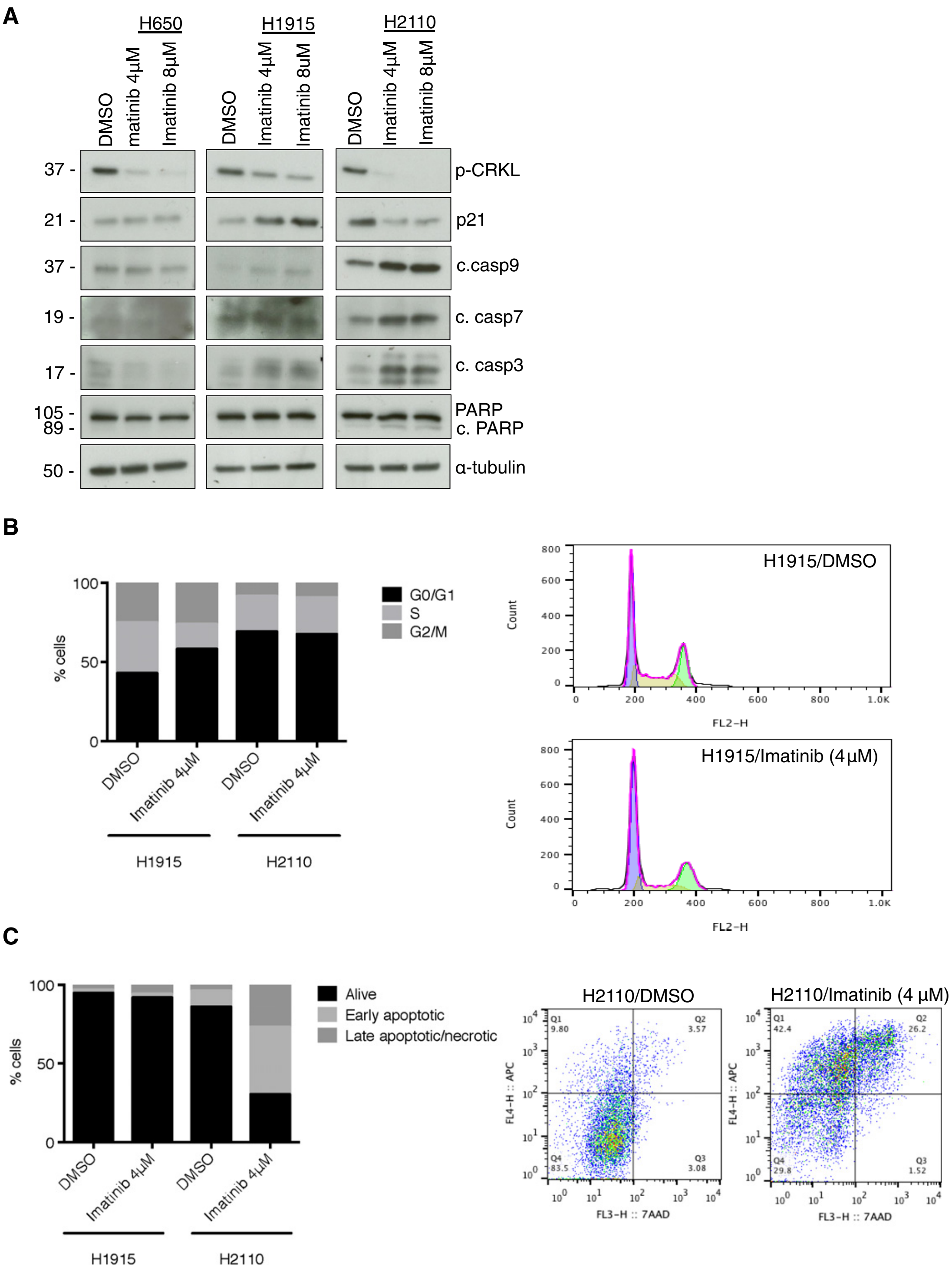

Figure EV4. Mechanism of imatinib-mediated toxicity in H1915 and H2110 cells.

A Western blot showing the relative levels of p21, apoptotic markers (cleaved caspases 3, 7, and 9 and PARP cleavage) and CRKL phosphorylation after 7-days treatment with the indicated concentrations of imatinib. C. Imatinib induces cell cycle inhibition in H1915 cells and apoptosis in H2110 cells.

B, C Imatinib induces cell cycle inhibition in H1915 cells and apoptosis in H2110 cells. (B) Left, cell cycle phase distribution in H1915 and H2110 cells after 48-h treatment with 4 μM imatinib; right, representative H1915 cell cycle profile. (C) Left, apoptosis analysis in H1915 and H2110 cells by annexin V-7AAD staining after 7 days of treatment with 4 μM imatinib. Data are mean of live (annexin V^--$7AAD^-$), early apoptotic (annexin V^+-$7AAD^-$), and late apoptotic/necrotic (annexin V^+-$7AAD^+$) populations; right, representative histogram for H2110 cells showing increase in the annexin V^+ population.

 © 2016 The Authors

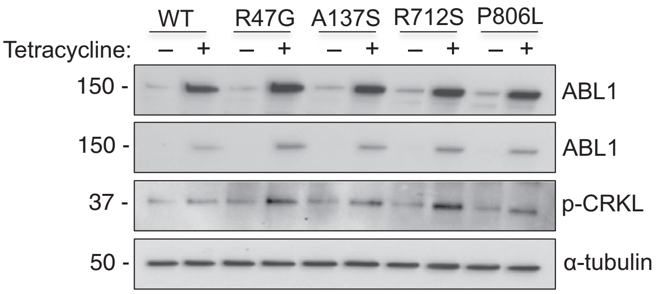

Figure EV5. ABL1 mutations found in primary lung tumors show increased activity toward CRKL when tested in Beas-2B cells.
Western blot analysis of relative levels of ABL1 variants and CRKL phosphorylation in Beas-2B cells with tetracycline-inducible expression of wild-type ABL1 and ABL1 mutants (R47G, A137S, R712S and P806L).

Source data are available online for this figure.

© 2016 The Authors
