# Supplementary figures and images for: Somatically mutated ABL1 is an actionable and essential NSCLC survival gene
Source: EMBO Mol Med. 2016 Jan 12;8(2):105–16. doi: 10.15252/emmm.201505456 (PMC4734836; doi:10.15252/emmm.201505456)

## Source Data for Figure EV2C

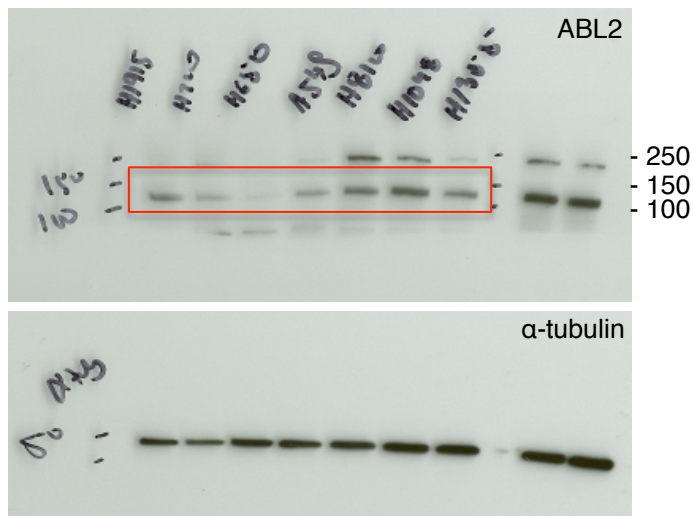

## Source Data for Figure EV2D

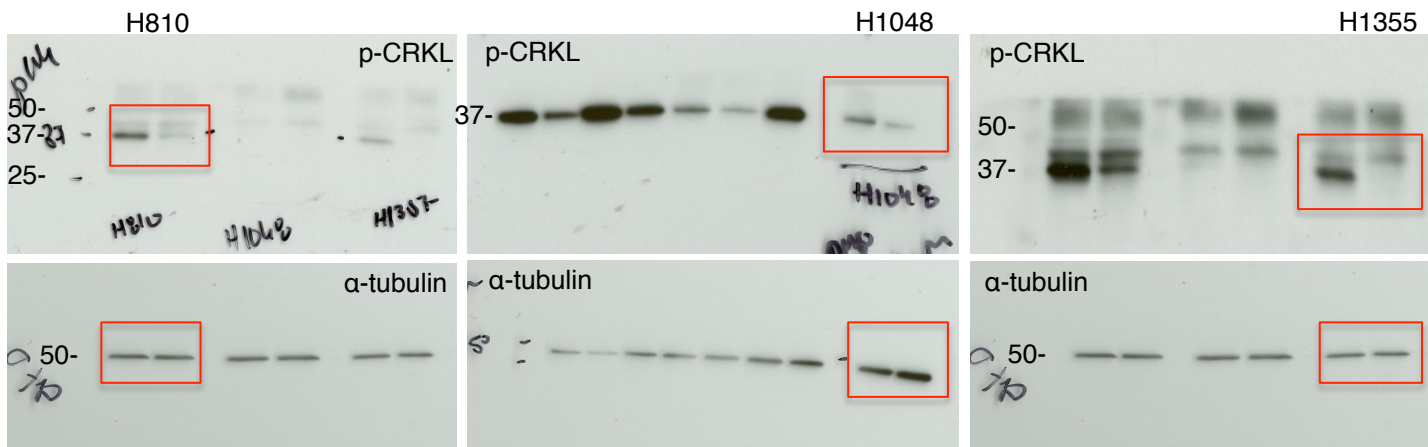

Supplement: Supplementary file 4 — Source Data for Expanded View [file EMMM-8-105-s007.zip › Source_Data_For_Expanded_View/EMM201505456V3_SourceDataForFigureEV2C-D.pdf]

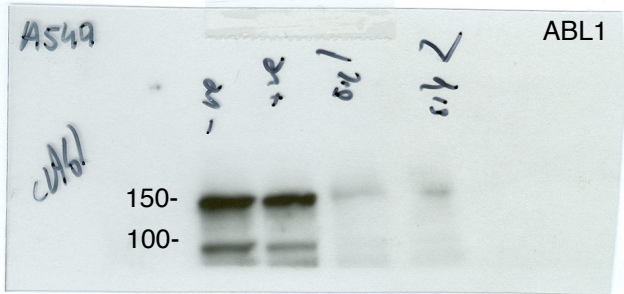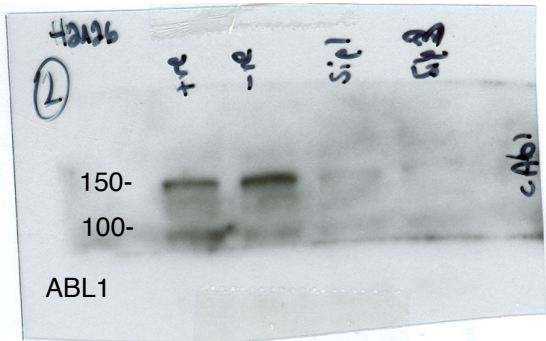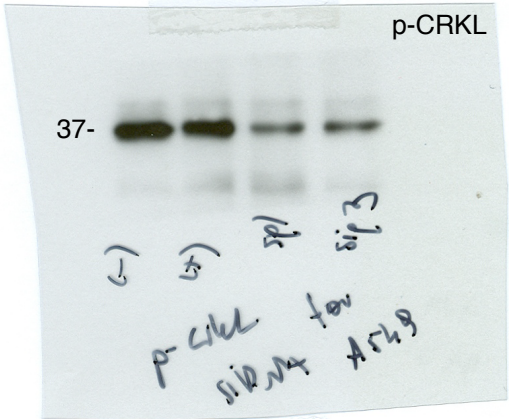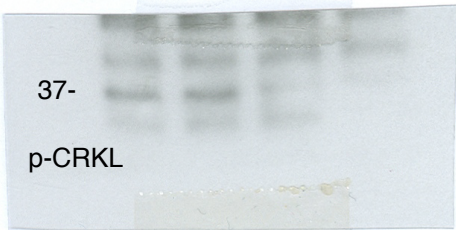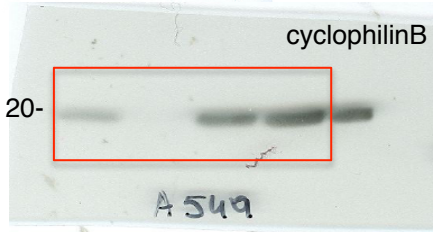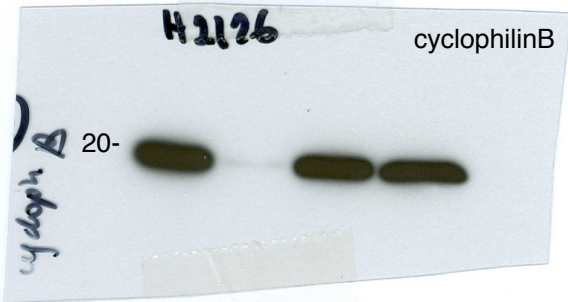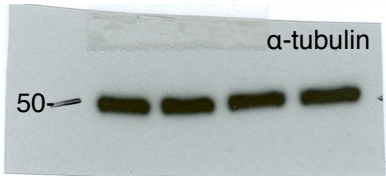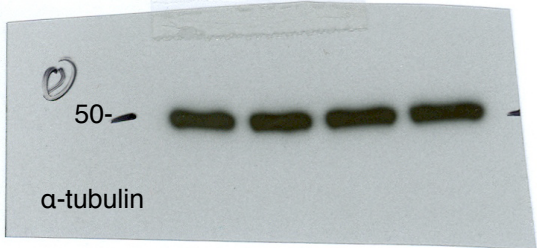

Supplement: Supplementary file 4 — Source Data for Expanded View [file EMMM-8-105-s007.zip › Source_Data_For_Expanded_View/EMM201505456V3_SourceDataForFigureEV3/EMM201505456V3_SourceDataForFigureEV3A.pdf]

Source Data for Figure EV3B

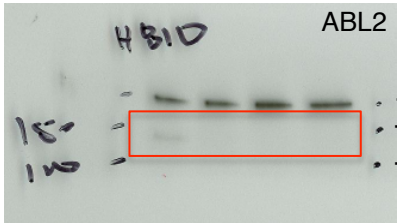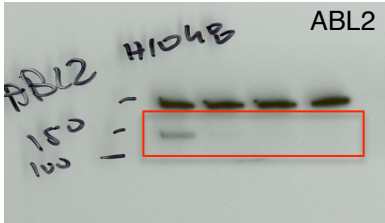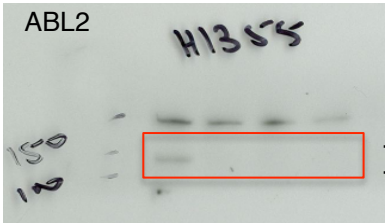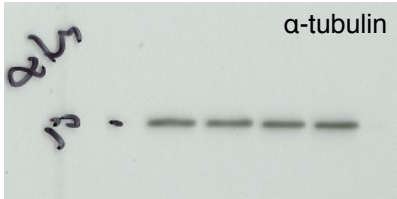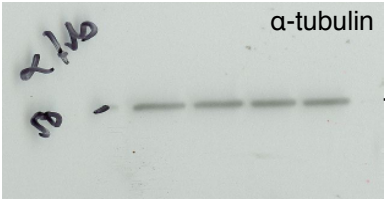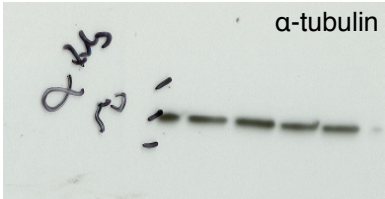

Supplement: Supplementary file 4 — Source Data for Expanded View [file EMMM-8-105-s007.zip › Source_Data_For_Expanded_View/EMM201505456V3_SourceDataForFigureEV3/EMM201505456V3_SourceDataForFiguresEV3B.pdf]

Source Data for Figure EV5

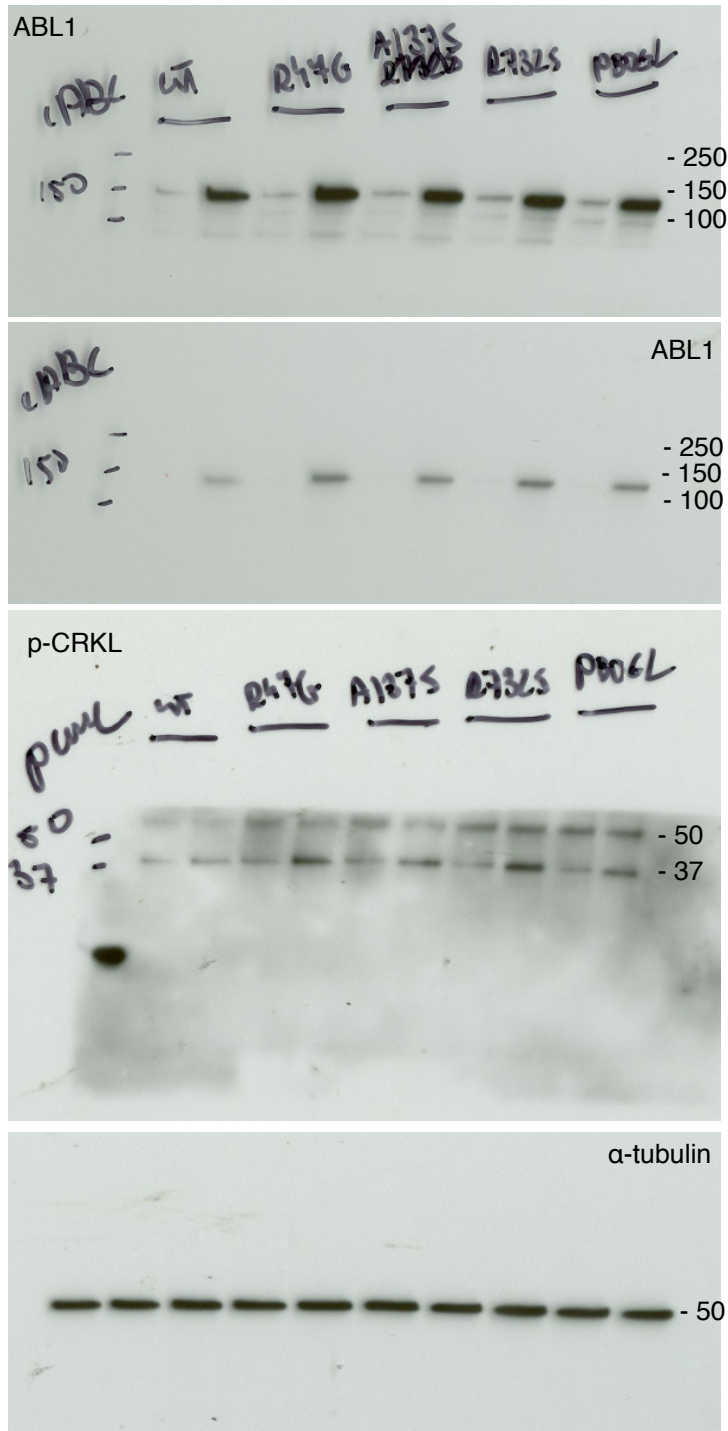

Supplement: Supplementary file 4 — Source Data for Expanded View [file EMMM-8-105-s007.zip › Source_Data_For_Expanded_View/EMM201505456V3_SourceDataForFigureEV5.pdf]

Representative Source Data for Figure 1B

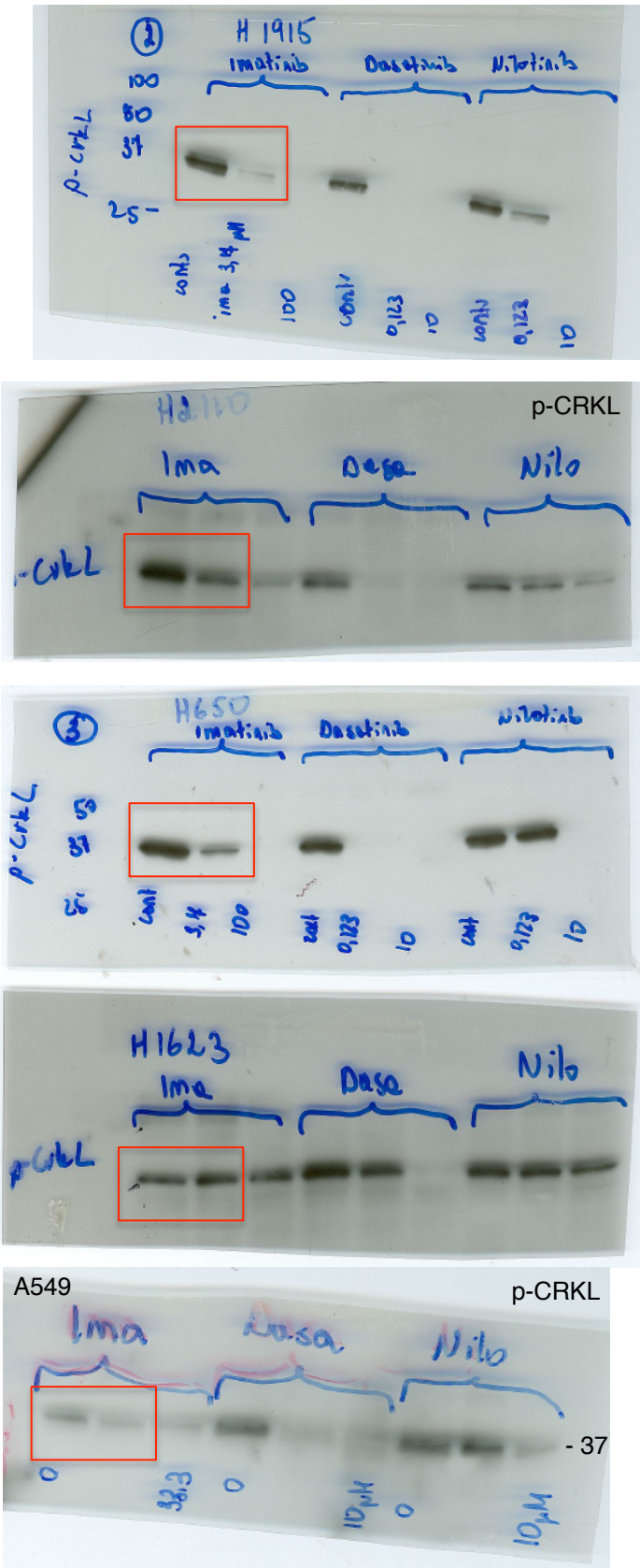

### Representative Source Data for Figure 1D

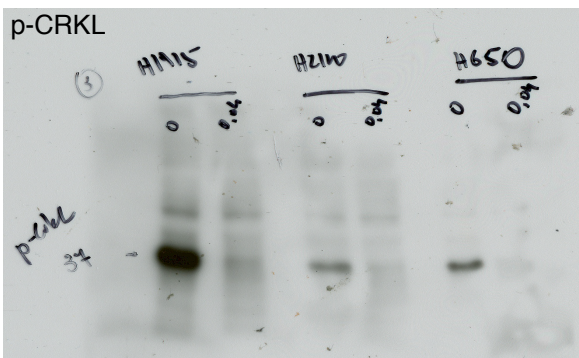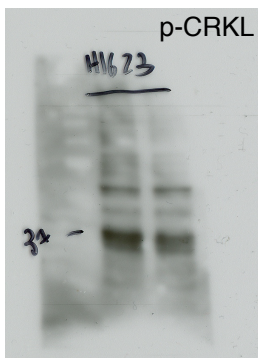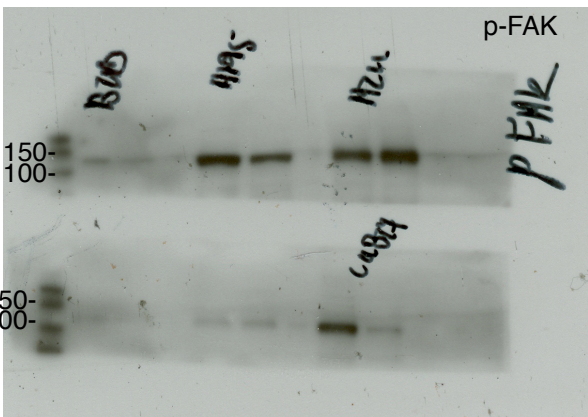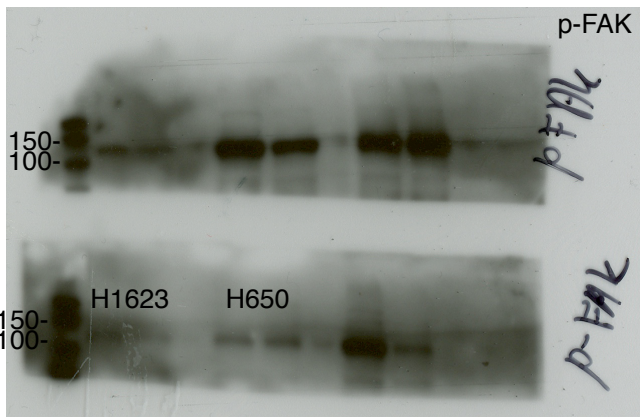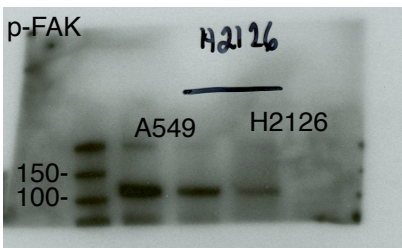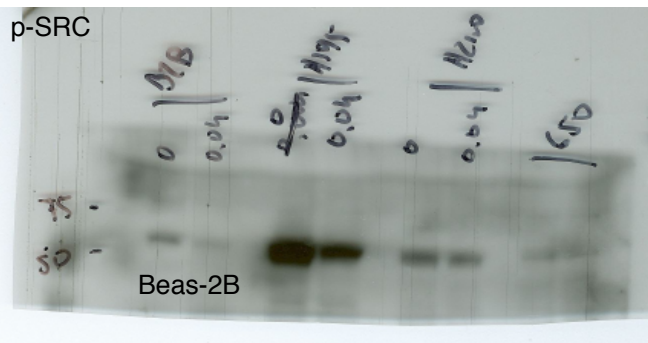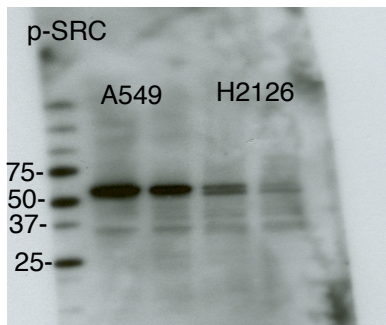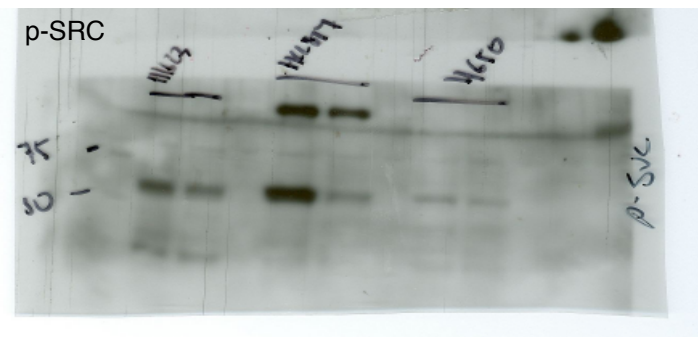

Supplement: Supplementary file 6 — Source Data for Figure 1 [file EMMM-8-105-s003.pdf]

Source Data for Figure 4E

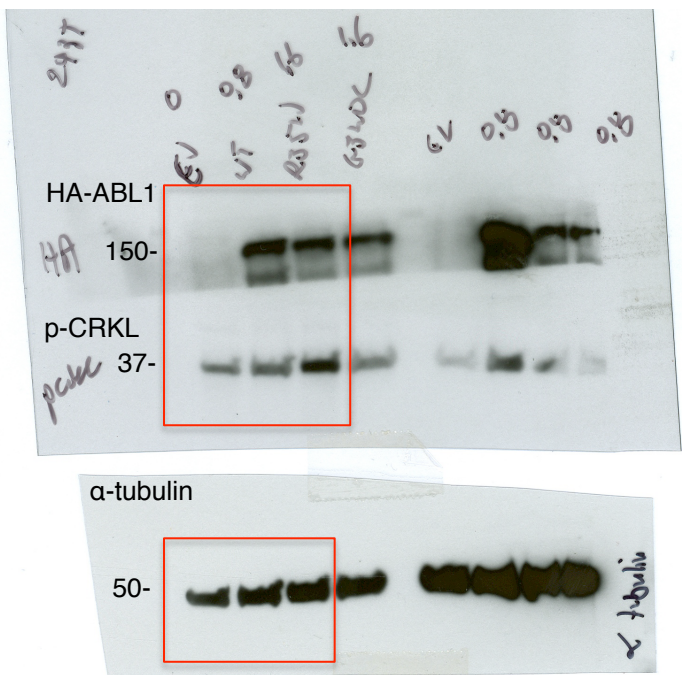

Source Data for Figure 4F

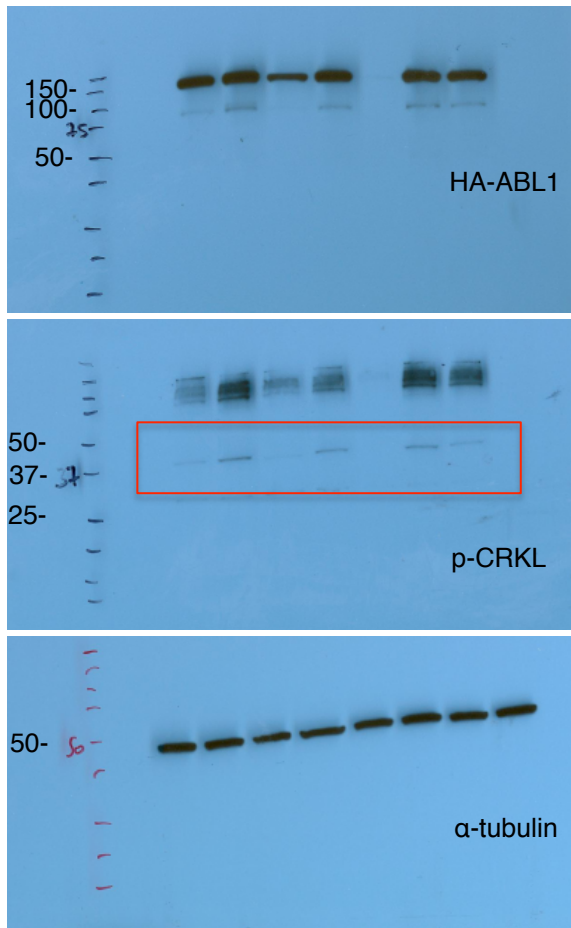

Supplement: Supplementary file 8 — Source Data for Figure 4 [file EMMM-8-105-s005.pdf]
