# Supplementary material for: Somatically mutated ABL1 is an actionable and essential NSCLC survival gene
Source: EMBO Mol Med. 2016 Jan 12;8(2):105–16. doi: 10.15252/emmm.201505456 (PMC4734836; doi:10.15252/emmm.201505456)

Source Data for Figure 2A

Dasatinib

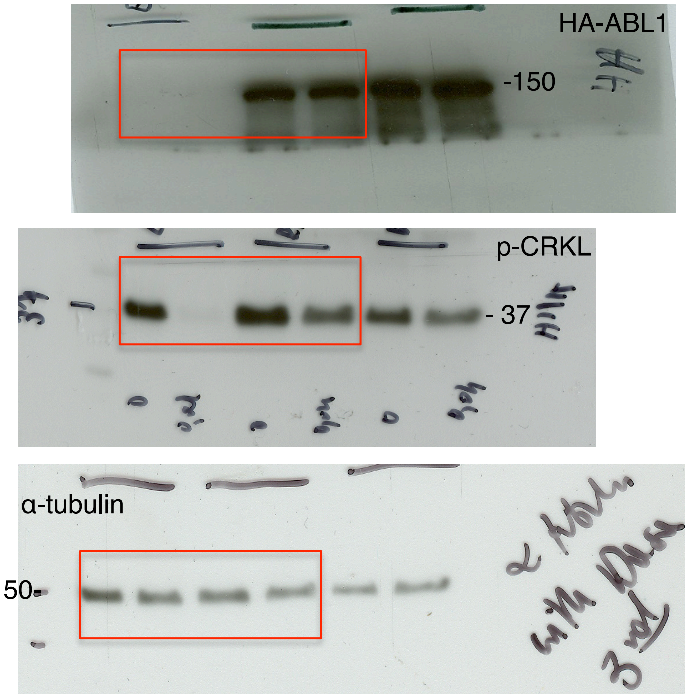

Imatinib

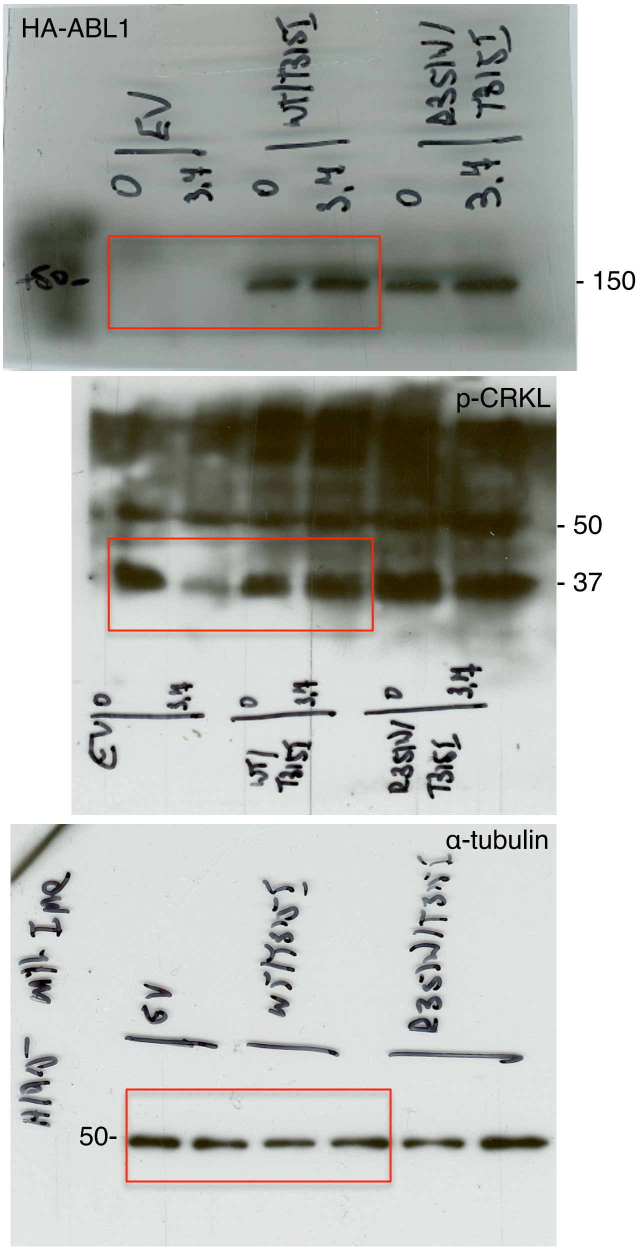

Source Data for Figure 2B

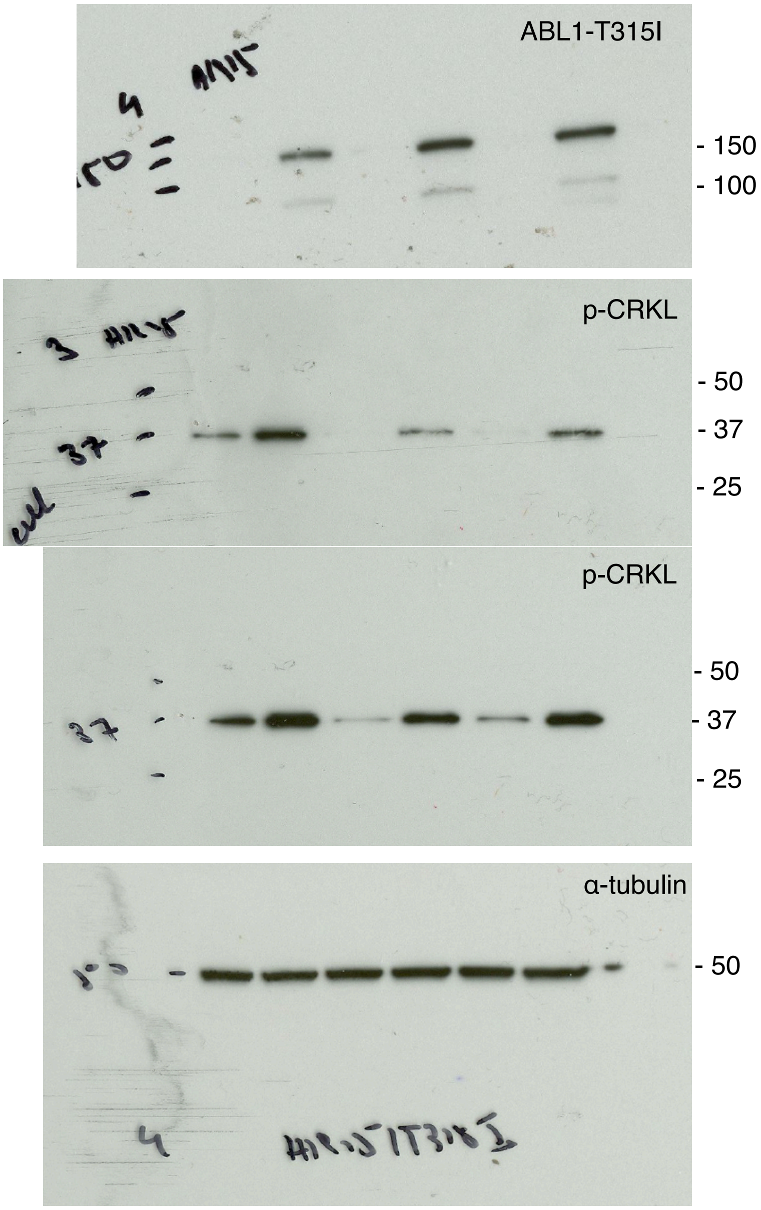

Source Data for Figure 2D

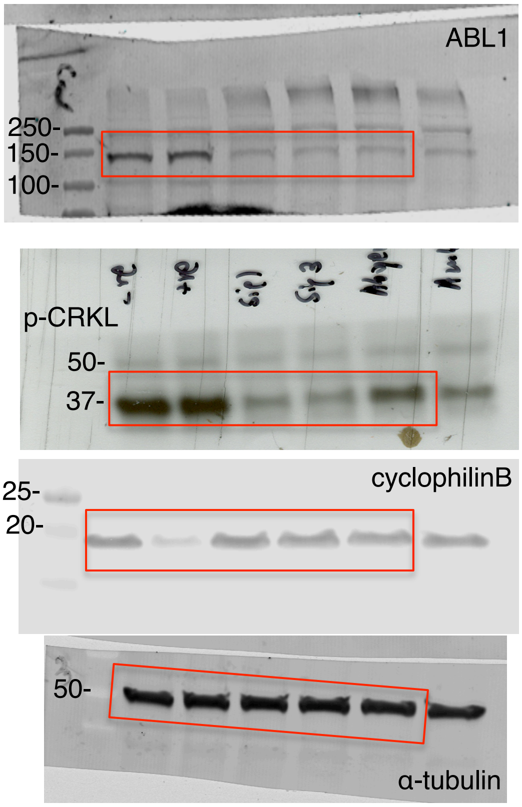

Source Data for Figure 2E

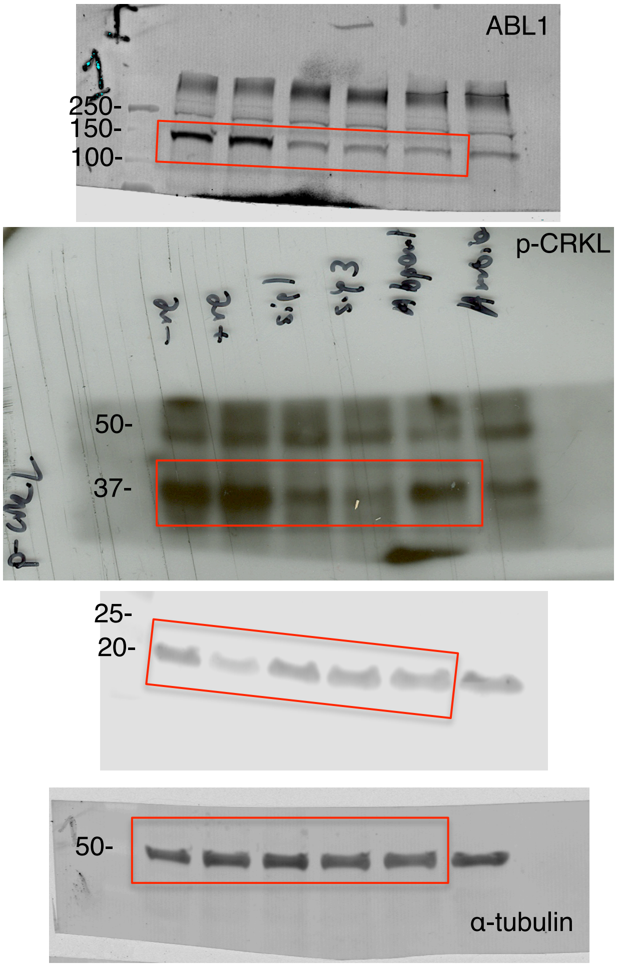

Source Data for Figure 2F

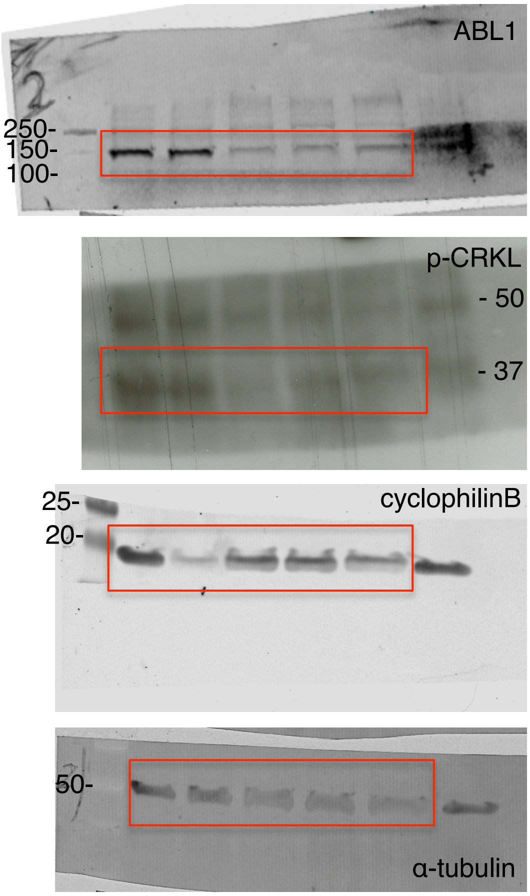

Supplement: Supplementary file 7 — Source Data for Figure 2 [file EMMM-8-105-s004.pdf]
